# New Insight into the Concept of Carbonization Degree in Synthesis of Carbon Dots to Achieve Facile Smartphone Based Sensing Platform

**DOI:** 10.1038/s41598-017-11572-8

**Published:** 2017-09-08

**Authors:** Zeinab Bagheri, Hamide Ehtesabi, Moones Rahmandoust, Mohammad Mahdi Ahadian, Zahra Hallaji, Farzaneh Eskandari, Effat Jokar

**Affiliations:** 1 Protein Research Center, Shahid Beheshti University G.C., Velenjak, 1983969411 Tehran, Iran; 2 Institute for Nanoscience and Nanotechnology, Sharif University of Technology, 1458889694 Tehran, Iran; 3 Department of Applied Chemistry and Institute of Molecular Science, National Chiao Tung University, 1001 Ta–Hsueh Road, 30010 Hsinchu, Taiwan

## Abstract

Direct pyrolysis of citric acid (CA) has been proved to be a facile bottom–up technique for making pristine carbon dots (CD) with homogenous size distribution. However, limited reports are available on systematic optimization of carbonization degree. In this investigation, pyrolysis temperatures between 160 °C and 220 °C were studied, based on CA thermal decomposition path, using various heating durations. The effect of the formation of more carbonized carbon particles (MCCPs), as the major byproduct of this method, on photoluminescence properties of CDs was also considered. The NaOH amount that neutralizes the solution and the effect of dilution on the emission intensity, were introduced as simple and accessible factors for monitoring carbonization degree, and an estimate of MCCP/CD ratio, respectively. The results show that the CDs fabricated at 160 °C, 50 minutes attain almost twice higher quantum yield (QY) of 29% than highest QY reported based on pyrolysis of CA. The so–prepared CDs can be employed as excellent candidates for turn–off sensing. As a proof of concept, detection limit of 50 nM for Hg^2+^ was achieved using a facile and inexpensive smartphone set–up that is able to quantify and compare fluorescent intensity in several samples simultaneously.

## Introduction

Chemical stability, low toxicity, and stable photoluminescence (PL) properties observed in Carbon dots (CDs), due to quantum confinement and edge effects, makes these zero–dimensional nanomaterials suitable for many advanced applications ranging from optical sensing and bioimaging, to fabrication of optoelectrical devices^1^. Since 2010, various top–down and bottom–up techniques have been offered to synthesize the nano–sized few layered graphenes, which include pyrolysis^2^, acidic oxidation^3, 4^, hydrothermal techniques^5, 6^, electrochemical techniques^7^, exfoliation based techniques^8–10^ and some other techniques^11–15^. Depending on the opted method, different size distributions, emission intensities and colours were reported for CDs. As introduced initially by Dong *et al*. in 2012, among the available fabrication techniques, pyrolyzing citric acid (CA) showed to be a facile bottom–up technique to achieve pristine CDs in aqueous solution. Table 1 shows a brief overview of employed pyrolysis techniques in which citric acid was used as precursor, providing information about the major characteristics of CDs including diameter, excitation and emission wavelengths (*λ*) and quantum yield (QY) values, where reported^2, 16–23^.

**Table 1:** Blue–emitting CD synthesis approaches, using citric acid as precursor.

Temperature (°C)	Time (min)	Diameter (nm)	*λ*_Excitation_ (nm)	*λ*_Emission_ (nm)	QY (%)	References
180	20	0.7–1.0	365	460	10%	18
	2400	3.5–14.3	360	460	2.6%	23
200	30	0.5–2.0	362	460	10.5%	2
		3.0–7.0	400	470	9%	16
		0.6–1.1	365	450	15.4%	19
		0.7–1.0	365	460	10.5%	18
		20.0	420	470	—	20
		2.3	365	460	—	24
	20	0.7–1.0	365	460	8.5%	18
260	40	9.0	362	460	—	17
	50	—	360	460	—	21
	17	—	360	460	—	22
270	20	2.0–10.0	365	460	2%	18

As it is observed in Table 1, since its invention, various citric acid pyrolysis approaches have been employed to produce high performance CDs. Dong *et al*. heated CA at 200 °C for 30 minutes to stablish this method. He also reported fabrication of graphene oxide (GO) particles by heating CA at the same temperature for longer duration of 120 minutes. Since elemental analysis of the products showed higher content of carbon in the so–called GOs, compared to CDs and CA, respectively, they named the process as “carbonization” and indicated that heating CA for longer duration at constant temperature, leads to higher carbonization degree and formation of GO^2^. Later, Wu *et al*. employed the same technique to make CDs by heating CA at 260 °C for 40 minutes and used the produced solution for detection of biothiols^17^. Huang *et al*. used 260 °C heating temperature with various heating durations^21, 22^.

In 2015, Wang *et al*., tried various synthesis conditions and reported valuable information on the topic^18^. They tried different heating temperatures and durations and analysed obtained CDs with various characterization techniques, reporting formation of larger carbon particles at higher temperatures and longer heating durations, which leads in excitation dependant PL emission. Systematic study about the influence of thermal decomposition conditions on the properties of the resulted quantum dots has been also done on a mixture of citric acid (CA) and ethanolamine (EA) in 2012^25^. Krysmann *et al*. investigated formation mechanism of CDs, or as they call them carbogenic nanoparticles, to achieve an optimum temperature. They concluded that PL behaviour of CDs strongly depends on the synthesis conditions and the products obtained at different pyrolysis conditions, in terms of time and temperatures, may belong to different material species, showing different PL properties. To best of our knowledge, however, there are no reports on employing a systematic approach to study the effect of heating time and temperature on PL properties and sensing ability of carbon–dots from CA source, as the only precursor. Specifically, synthesis according to the thermal decomposition path of CA has not been considered. Furthermore, attention to the formation of more carbonized carbon particles (MCCPs), as the other byproduct of pyrolysis of citric acid, has not been considered as a factor that would be misleading due to the changes it imposes to the PL properties of the solution.

In this investigation, an experiment plan was arranged in order to obtain a comprehensive insight of the influence of synthesis conditions on optical properties of resulted CDs. Various characterization techniques were employed to study different properties of the resulted CDs and to converge to the best optimized synthesis condition. The NaOH amount used for neutralizing the solution, was introduced as a simple and accessible factor for monitoring the degree of carbonization^26–29^. Furthermore, the MCCP/CD ratio was estimated by considering the effect of dilution on the emission intensity. Since detection of even traces of heavy metal ions, like mercury ion, is of utmost importance, due to environmental contamination and human health issues, this investigation focuses on employing inexpensive, accessible and fast techniques to produce mix–and–detect turn–off sensor, for detection of very low concentrations of Hg^2+^ ion using smartphone application. Common optical studies were employed to confirm the capability of the employed image processing smartphone application. Furthermore, the technique is able to quantify and compare the amount of fluorescent intensity in several samples at the same time, which can be considered as a significant advantage.

## Methods

### Chemicals

For the purpose of CDs synthesis and analysis, anhydrous citric acid, sodium hydroxide, quinine sulphate, sulfuric acid, phosphate buffered saline (PBS) tablet and mercuric chloride were purchased from Merck. The chemicals were all analytically pure and used as received. Milli–Q water at room temperature was used for diluting samples to the desired concentration throughout the experiment.

### Synthesis of CDs

As mentioned earlier, pyrolyzing CA was employed for synthesis of CDs. One gr of anhydrous citric acid was used as precursor and heated at the assigned temperature and duration^2^. The achieved liquid was neutralized using 0.5 M NaOH solution to pH 7.0, under vigorous stirring condition and the NaOH volume used was recorded for all samples. In this experiment, various fabrication conditions were selected to be investigated. The opted temperatures and heating durations were assigned based on triangulation of preliminary studies on the decomposition temperature of CA, the effect of synthesis conditions on the optical properties of the resulted CDs, and available data in literature.

### Characterization

To obtain information about thermal decomposition path of CA, differential thermal analysis (DTA) and thermal gravimetry (TG) curves were obtained in air atmosphere, at a heating rate of 20 K/min (Linseis STA PT1600, USA). Chemical bond analysis was performed using Fourier transform infrared spectroscopy (FTIR) (Bruker Optics, Germany) and CHNS–O elemental analyser (Costech Elemental Combustion System, ECS 4010, Italy) was used to determine carbon and hydrogen content of various samples.

The obtained CDs were then characterized by Dynamic light scattering (DLS) (Nanophox, Sympatec GmbH, Germany) in the automatic mode, to determine particle size distribution, in the scattering angle of 90 degrees, using HeNe laser light source, with a maximum intensity of 10 mW and wavelength of 632.8 nm.

Transmission electron microscopy (TEM) (Zeiss – EM10C, Germany), operated at 80 kV. Analysis was performed to confirm morphological characteristics of the quantum dots. Samples were prepared for TEM by adding a drop of nanoparticle suspension to a Formvar carbon coated copper after sonication (Misonix– S3000, USA). Further details of the height of the CDs were obtained using atomic force microscopy (AFM) in non–contact mode, using VEECO multimode scanning probe microscope, USA. AFM images were analysed with WSXM software provided by Nanotec Electronica^30^.

The optical properties were analysed by ultraviolet–visible (UV–vis) spectroscopy (PerkinElmer’s LAMBDA 950 UV/Vis/NIR Spectrophotometer, USA), utilizing quartz cuvettes with optical path lengths of 10 mm. Photoluminescence and photoluminescence excitation (PLE) spectra were obtained using a spectrophotometer (Varian Cary Eclipse Fluorescence Spectrophotometer, Agilent, USA).

### Mercury ion detection approach

Carbon dot solutions were prepared with 0.15 optical density (OD) at 360 nm absorption wavelength. Mercury ion stock solution was prepared by dissolving HgCl_2_ in PBS buffer solution (10 mM, pH 7.4) at room temperature. Two approaches were then employed to determine the influence of adding Hg^2+^ to the CD solution. Low–volume high–concentration of mercury ion was used to approximately keep the OD of the solution constant. First, titration technique was employed for immediate recording of the quenched fluorescence spectra of CD solution, with step–wise increase of Hg^2+^ ion concentration in fluorometer quartz cuvettes. In the other technique, 0.05, 0.1, 1, 2, 5, 10, 20, 50, 100 and 200 µM of Hg^2+^–CD solutions were prepared and the emission intensities of the samples were observed by naked–eye and recorded using smartphone, in an experimental set–up that will be described in the next section. For the purpose of selectivity test, HgCl_2_, FeSO_4_.7H_2_O, CaSO_4_.2H_2_O, ZnCl_2_, CuCl_2_.7H_2_O, MgSO_4_, and Pb(CH_3_COO)_2_.3H_2_O were added into the CD dispersions to make equal cation concentrations of 100 µM.

### Smartphone–based detection

**Figure 1:**
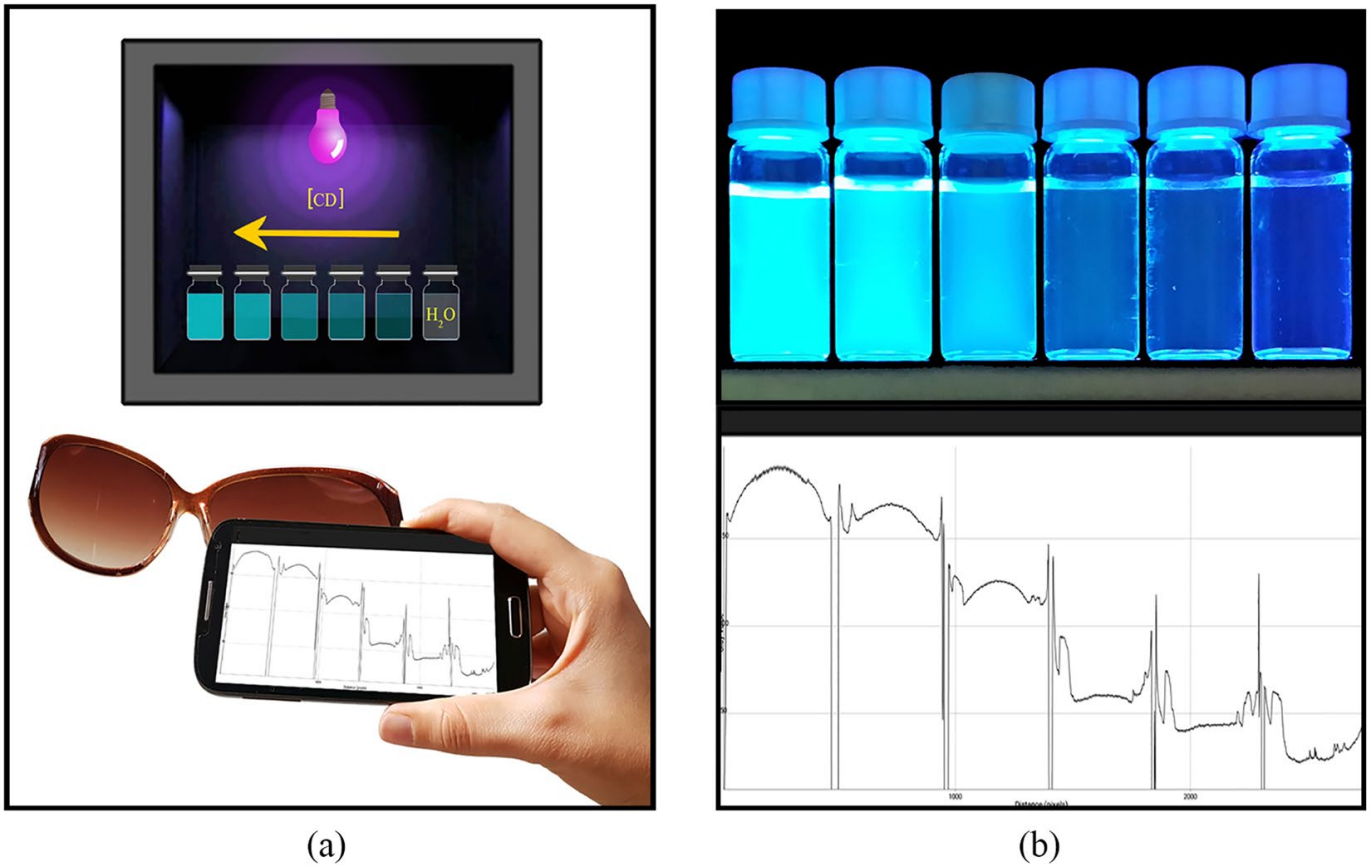
(**a**) Schematic representation of the smartphone fluorometer set–up, (**b**) Mobile phone image example and the corresponding IJ–mobile intensity spectra.

In order to achieve a simple and inexpensive quantitative measurement tool for detection of heavy metal ions, an android free application (IJ_Mobile application, version 1.1, 2013), was employed^31^. To set–up a smartphone fluorometer, as shown in Fig. 1(a), a standard gel document instrument was used as UV radiation–chamber. The instrument is equipped with five 8.0 W, 365 nm, UV tube lamps, providing a uniform intensity distribution of appropriate wavelength, and a 365 nm band pass filter with the 20 nm of full–width at half maximum (FWHM). After putting the samples inside UV–chamber, mobile phone camera with 13 MP resolution was employed to capture images. In order to block background light, a good image capturing angle should be selected along with typical sunglasses, as 410 nm high pass UV filter. The captured images were then processed using *plot* option in IJ_Mobile application, in which the emission intensity from various samples can be measured and compared adequately at the same time. Figure 1(b) represents an example of the mobile phone image and the related IJ–mobile plot of the intensity spectra.

## Results and Discussion

### Synthesis of CDs

**Figure 2:**
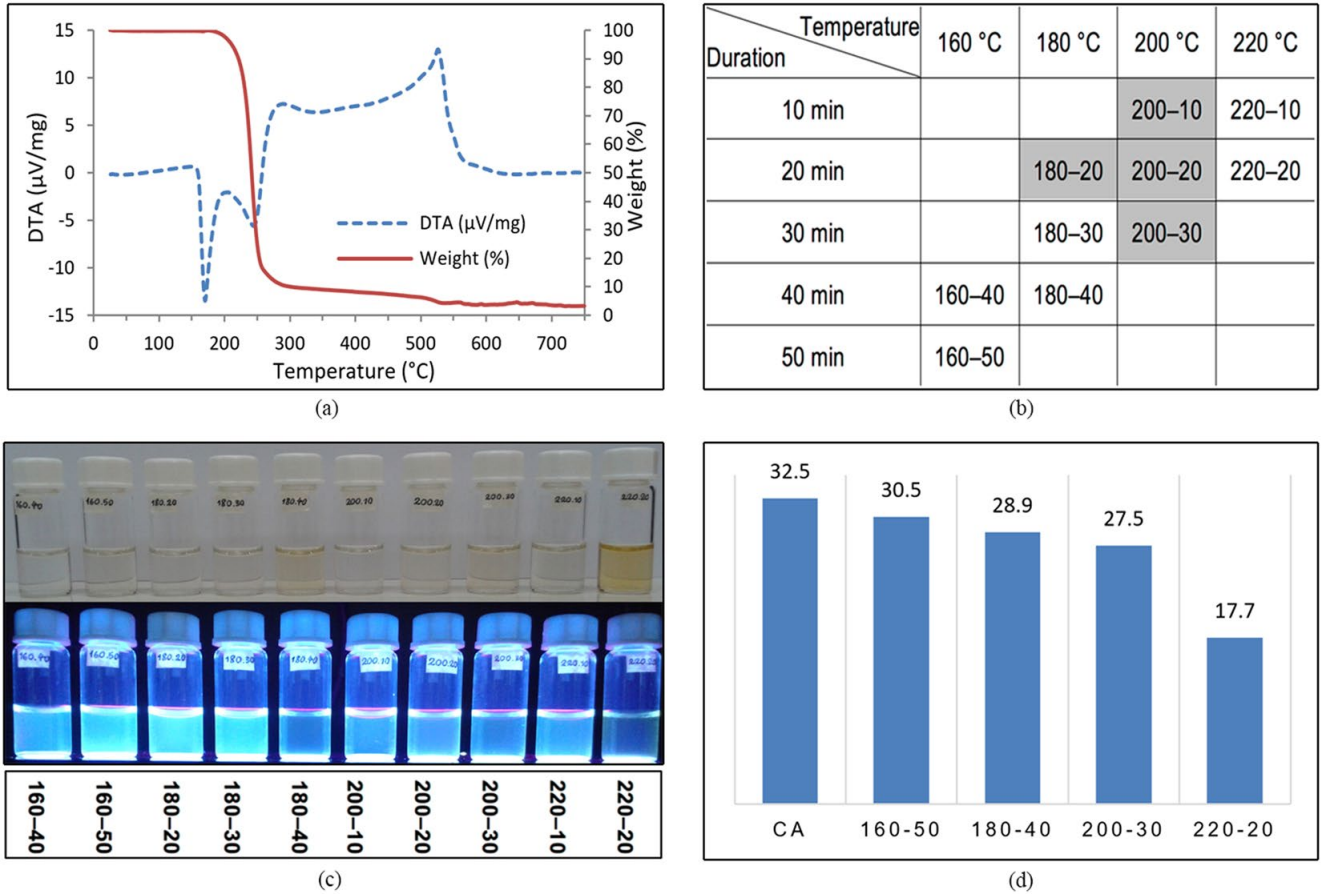
(**a**) DTA and TG curves of CA, (**b**) Table of samples that are fabricated, (**c**) Samples under visible light and UV radiation, (**d**) Amounts of 0.5 M NaOH used for neutralization in ml.

In this investigation, a systematic synthesis study was performed. Synthesis temperature interval was selected based on a thermal decomposition path of CA and the heating durations were also opted based on a preliminary studies on the optical properties of the resulted CDs, as well as available data in literature^2, 16–23^. As indicated in Fig. 2(a), DTA of citric acid reports a strong endothermic peak at around 160 °C implying that CA changes its composition at this temperature. On the other hand, the TG curve shows weight loss initiation at around 160 °C, climaxing at around 220 °C. Hence, based on the change in the physical state of CA, as revealed by the two peaks in the DTA curve, an insight on the process of decomposition was obtained. Pyrolysis of citric acid was investigated between 160 °C to 220 °C. The distribution of the 10 samples chosen to be fabricated are shown in Fig. 2(b). The names of the samples reflect the heating temperature and duration, one after the other; e.g. 180–30 has been heated at 180 °C for 30 minutes. The highlighted cells are samples that has been synthesized in previous studies. During synthesis, despite previous reports about observing an orange colour melted CA liquid^2^, as observed in Fig. 2(c), in many of the samples with strong fluorescent intensities, the liquids colour did not turned into orange at all.

In this investigation, as shown in Fig. 2(d), for pure anhydrous CA, 32.5 ml NaOH 0.5 M was required for neutralization. As different amounts of NaOH were neutralizing different samples, in order to have equal volumes for all samples, they were all brought to an identical volume, equal to 32.5 ml by Milli–Q water.

### Photoluminescence properties

**Figure 3:**
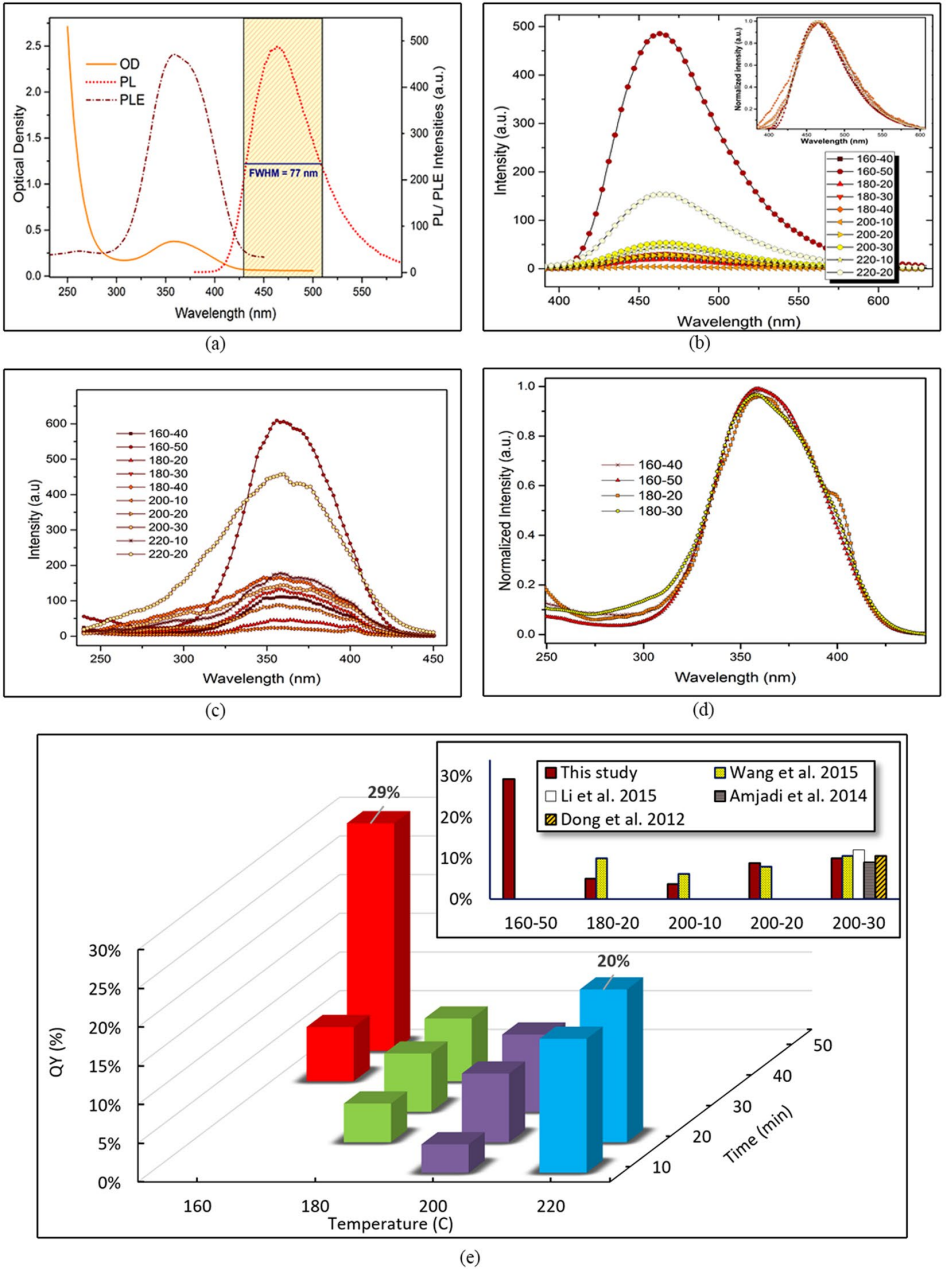
(**a**) PL, PLE and OD peaks, (**b**) PL of the diluted samples (inset: normalized peaks), (**c**) PLE diagram of all samples, (**d**) normalized PLE peaks showing narrower peaks only, (**e**) QY based on heating temperature and duration (inset: comparison of highest achieved QY in previous studies).

Various investigations have concentrated on explaining the PL mechanism in pristine and defected CDs, with different chiralities, shapes, surface structures and sizes. The phenomenon was first considered to be originated from free zigzag sites, due to the carbene–like triplet ground states^5^. Later, more comprehensive mechanisms were proposed, explaining intrinsic reasons behind PL in pristine CDs, as well as introducing the effect of defect in the exhibited PL emission^32^. In pristine CDs, the phenomenon is known to be due to the electron, or charge transfer between π and π* energy levels, as a result of quantum confinement effect^33^. The domain size or the number of fused aromatic C–C rings however, play an important role in the energy gap between π and π*, leading to different emission colours and properties^34^. Other than the actual size of the CDs, existence of different functional groups on the surface of the CDs, such as carboxyl groups, hydroxyl groups, amino groups, etc. would influence the electric charge distribution on CDs and formation of smaller *sp* ^2^ domains on the surface of the CDs, which in–turn grants PL emission^35^. In this study, as shown in Fig. 3(a), the so–prepared CDs show a UV–vis spectrum with maximum absorption wavelength at 360 nm. The PL intensity diagram of CDs, with an excitation wavelength of 360 nm, shows blue emission wavelength of 460 nm. The FWHM below 100 nm admits a homogeneity in size distribution of the nanoparticles^36^.

Figure 3(b) illustrates the photoluminescent emission of the samples diluted with 1/16 ratio to minimize reabsorbance effects^37^, as much as possible. The normalized peaks in the inset declares the similarity of the PL trend in all samples, as well as their equal maximum emission wavelength, i.e. 460 nm. As the image implies, 160–50 sample acquires quantitatively the highest emission intensity at low concentration, compared to other samples. Figure 3(c) demonstrates the PLE spectrum of all samples, showing a narrow PLE peak with highest intensity again in 160–50. The PLE peaks were then normalized and as Fig. 3(d) shows, samples heated at lower temperatures for shorter periods, namely the 160–40, 160–50, 180–20 and 180–30 samples, all obtain narrow PLE peaks, confirming more homogeneity in terms of size distribution in these samples^38^.

Quantum yield, as a major value that reflects the quality of the synthesized samples was also obtained for all solutions, in ODs below 0.1 with excitation wavelength of 360 nm^37^. The 1/16 diluted CD solutions were employed for calculation of QY, using quinine sulphate as standard. The refractive index is equal to 1.33 for both the samples and the reference solutions. In Fig. 3(e), QY of all fabricated samples versus heating temperature and duration is demonstrated and the highest achieved QY of this study was compared with data from previous studies. The highest quantum yield is observed in 160–50 sample, with QY value equal to 29%, however, the QY of 220–20 is also clearly higher than values reported previously in literature. Previous experimental investigations were majorly focused on 200 °C and higher heating temperatures. The relatively high QY of 160–50 sample implies that the CDs are well surface–passivated, most likely by the incompletely carbonized CA molecules, as well as by carboxyl and hydroxyl groups on the edges of the quantum dots^39^.

### Factors influencing photoluminescence intensity

**Figure 4:**
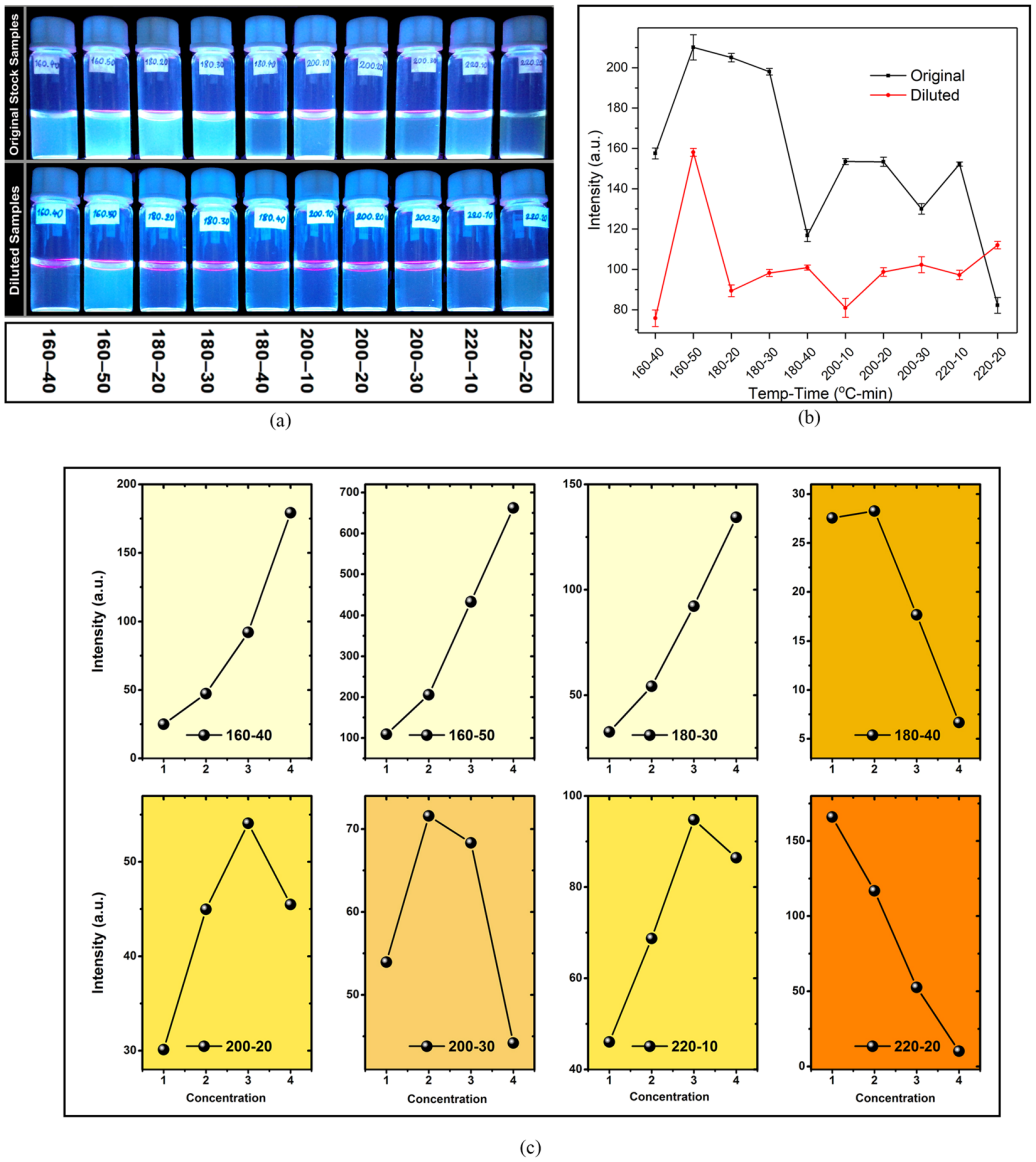
(**a**) Samples under UV light in original (up) and diluted (down) states, (**b**) Emission intensities of samples in original and diluted states, (**c**) The effect of gradual dilution on the fluorescent intensity.

Figure 4(a), shows the samples under UV radiation in both original and diluted conditions. Using smartphone fluorometer, the emission intensities of the samples were recorded, as the diagram shows in Fig. 4(b). However, the process of diluting was observed to have a noticeably inconsistent effect on emission intensity in various samples. Figure 4(c) depicts the effect of dilution, from original sample to half, one–fourth, one–eighth and one–sixteenth, on the emission intensities of samples, in which similar trends were highlighted with identical background colours. In Fig. 4(c), numbers from 1 to 4 indicates a stepwise increase in the concentration of the CD solution, which leads to remarkably different trend in emission intensity of various samples.

Seeking to elucidate the reason behind these various trends, the process of CD formation was reviewed. As a result of direct pyrolyzing CA, two main products are achieved. Dong *et al*. claimed that having CA heated for longer durations, leads to higher carbonization degree, equal to formation of *GO* ^2^. On the other hand, Wang *et al*.^18^ reported formation of so–called *larger carbon particles* at higher temperatures and longer heating durations, which weakens the overall PL emission. Hence, in order to show that both temperature rise and longer heating duration leads to higher carbonization, elemental analysis was performed to compare the carbon content in 160–50, 220–20, 160–120 and 220–120 samples, as shown in Table 2.

**Table 2:** Elemental Analysis of 160–50, 220–20, 160–120 and 220–120 samples.

Sample	C (wt.%)	H (wt.%)	O (wt.%, calculated)
**160–50**	37.81	4.25	57.94
**160–120**	40.12	3.87	56.01
**220–20**	45.33	3.81	50.86
**220–120**	68.85	4.74	26.41

The MCCPs, on the other hand, seem to have quenching effect on PL of the CDs, leading to various trends in PL emission vs. concertation. Similar quenching phenomenon was reported about GO previously, that occurs due to the less possibility of π–π interaction between existing GO particles and CDs^40^.

In 160 °C and 180 °C heating temperatures, minimal formation of MCCPs grants a decrease of emission, as a result of dilution. At 200 °C, the heating duration shows itself as the influencing factor on the solution’s MCCP content, i.e. the higher the heating duration, the higher the MCCP content. The trend in finally saturated in 220–20 sample, in which there are more MCCPs in the solution than other samples. At this temperature, referring to DTA and TG curves, almost all of CA molecules would be pyrolyzed to either CD particles or MCCPs and the efficiency of the chemical reaction is high. Assuming that MCCPs have a quenching effect on fluorescent emission of CDs, as GO particles do, this quenching effect would be minimized as a result of diluting the solution and keeping the two particles far from each other.

**Figure 5:**
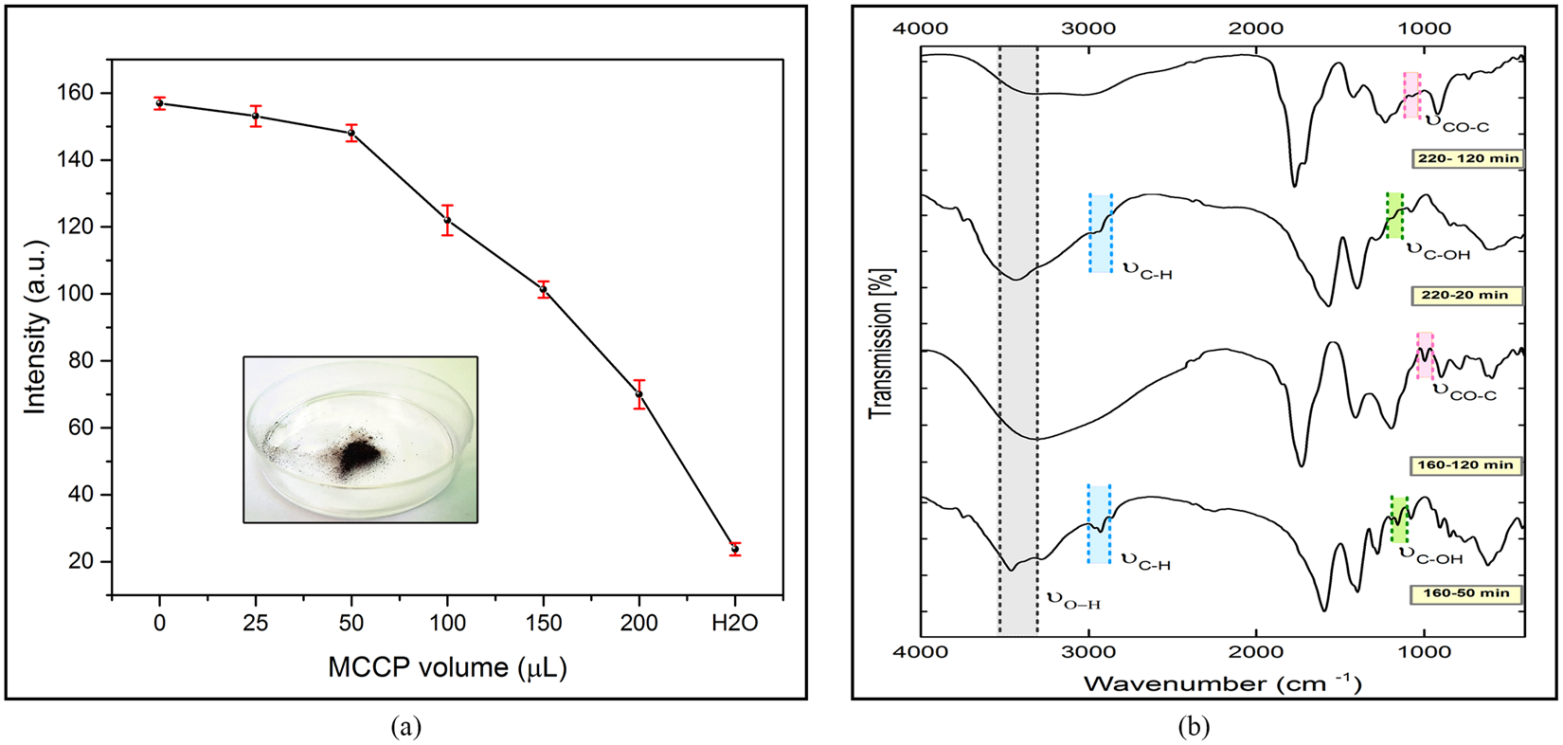
(**a**) The quenching nature of MCCPs, (**b**) FTIR spectra of 160–50, 220–20, 160–120 and 220–120 samples.

In order to show the quenching effect of MCCPs, another test was conducted. In this test, 160–50 was opted as the reference fluorescent sample and the probable quenching behaviour of MCCPs was tested by different amounts of 220–120 (10 mg/ml) to the solution, as illustrated in Fig. 5(a) (inset: 220–120 MCCP powder). The characteristic absorption patterns of chemical bonds were obtained, as provided in Fig. 5(b). FTIR of 160–50 and 220–20 show absorption of stretching vibration C–H suggesting that they still contain some incompletely carbonized CA, whereas in 160–120 and 220–120 samples, nearly no absorption is observed in C–H range, implying that the CA has been carbonized more completely. In addition, the C–OH group has changed to epoxy (CO–C) as a result of higher pyrolysis duration at both temperatures.

### CDs characterization

**Figure 6:**
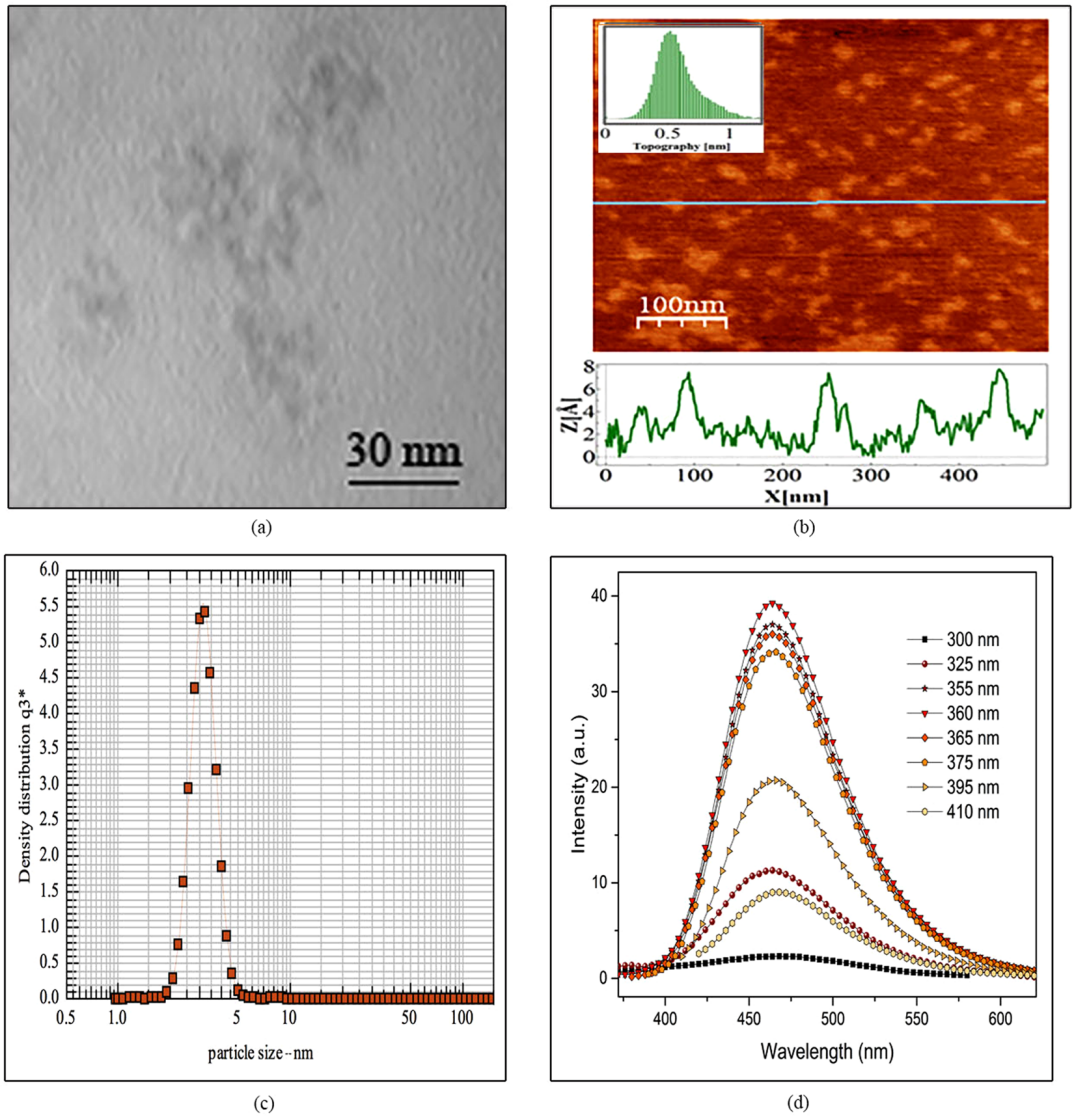
(**a**) TEM and (**b**) AFM images of as prepared GQDs, (**c**) DLS particle size distribution diagram, (**b**) Excitation independent emission of 160–50.

Figure 6 shows the characteristic analysis of 160–50. According to AFM and TEM results, Fig. 6(a) and (b), the diameters are mainly distributed in the range of 2 to 5 nm (3 nm average diameter). Based on the AFM results, topographic heights are mostly between 0.6 and 1.5 nm with the average height of 0.8 nm, suggesting the presence of 1 to 3 layers of graphene in each individual CD. DLS results show that the mean diameter of the CDs, Fig. 6(c), was obtained to be 3.1 ± 0.4 nm. The narrow peak shows a rather uniform size distribution in CDs.

Figure 6(d) shows that the emission wavelength of the so–prepared CD is independent from the excitation wavelength. Contradictory reports have been presented on the dependency of emission wavelength to the excitation in CDs. However, the content of GO, larger particles or MCCPs, as observed in experimental investigations^2, 18, 41, 42^ and theoretical studies^33, 43, 44^, plays an important role on dependency of the emission wavelength to the excitation.

### Quenching with mercury ion

Mercury ion is known to be capable of quenching the fluorescence emission of CDs^17, 19^. In fact, most of CD–based metal–ion sensors are functioning by PL turn–off observation. Lots of research has been conducted to explain the fluorescence quenching mechanism of metal ions, reporting that the phenomenon occurs due to the electron, charge or energy transfer resulted from CD–Hg^2+^ interactions. The carboxyl and hydroxyl functional groups, on the surface of CDs can selectively interact with the introduced metal ions, which subsequently changes the electronic structure of CDs and the distribution of excitons, leading to non–radiative recombination of the excitons through effective electron, charge or energy transfer and quenching of the fluorescence emission^45^.

**Figure 7:**
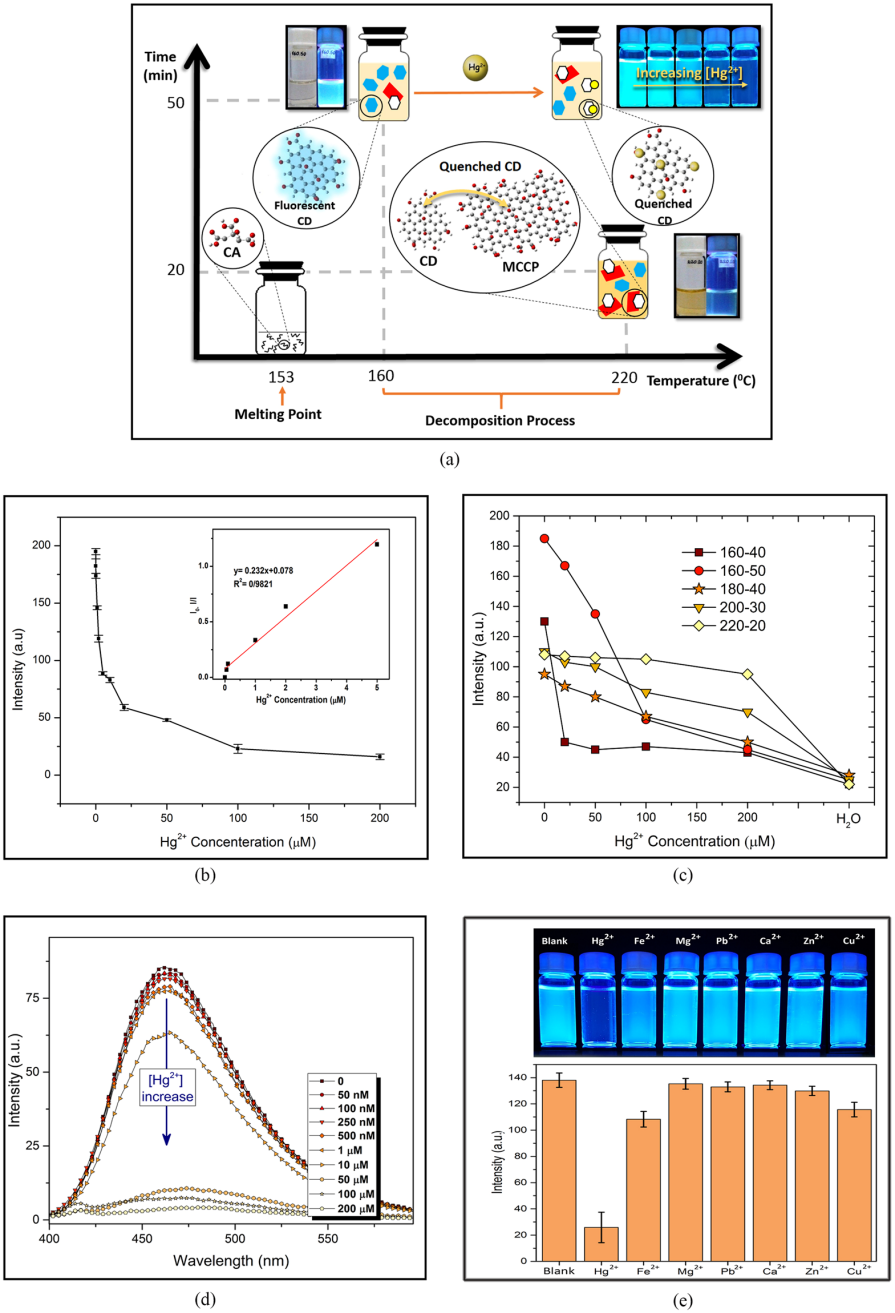
(**a**) Schematic diagram of CD/MCCP creation over temperature and time scales and the influence of the existence of Hg^2+^ ion and MCCP particles on quenching PL emission of CDs, The quenching effect of Hg^2+^ (**b**) on various samples using smartphone, (**c**) on 160–50 sample using spectrofluorometer (inset ion concentration log vs. PL intensity), (**d**) Quenching ability of 160–50 exposed to 100 µM of various cations under UV radiation.

The process of CD and MCCP creation over time and temperature scales is schematically depicted in Fig. 7(a). In addition, the influence of the existence of MCCPs and/or introducing Hg^2+^ ion on quenching the fluorescent emission of CDs are illustrated, where the blue and white hexagons represent fluorescent– and turned–off CDs, respectively, red rectangles denote for MCCPs and mercury ions are represented by yellow circles.

Initially, different synthesized samples were exposed to different concentrations of mercury ion and their response was observed and recorded using smartphone set–up. As plotted in the diagram of Fig. 7(b), 0, 20, 50, 100 and 200 µM of Hg^2+^ ion were added to diluted CD solutions. Due to initial stronger emission intensity, higher QY and less MCCP formation during fabrication process, 160–50 shows an apparently better PL intensity drop over each step of increasing Hg^2+^ concentration and hence, the sample can be employed reliably for detection of mercury ion. Then, as shown in Fig. 7(c), 0.05, 0.1, 1, 2, 5, 10, 20, 50, 100 and 200 µM of Hg^2+^ was introduced to 160–50 CD solution and studied using smartphone sensing platform. Quenching PL behaviour was linearly proportional to the concentration of Hg^2+^ over low concentration between 0 to 5 nM, with a detection limit of 50 nM, which is lower the previous report on Hg^2+^ detection, using smartphone app^46^. Spectrofluorometer studies, as observed in Fig. 7(d), shows that increasing the concentration of Hg^2+^, leads to the emission intensity drop without any shift in maximum emission wavelength. The selectivity diagram of 160–50 CD solution, quantified by smartphone app, in the presence of 100 µM of various metal ions is shown in Fig. 7(e), from left to right, blank CD solution, Hg^2+^, Fe^2+^, Mg^2+^, Pb^2+^, Ca^2+^, Zn^2+^, Cu^2+^.

## Conclusion

Carbon dots were fabricated by direct thermal decomposition of citric acid using a systematic approach to study the influence of synthesis conditions on PL properties of the resulted CDs, between 160 °C to 220 °C, over various heating durations. The CDs fabricated at 160 °C for 50 minutes attained very high fluorescent intensity, photostability over one month and a quantum yield of 29%, which is almost twice higher than the highest QY reported for CDs fabricated by the same technique.

Carbonization degree at various synthesis conditions was studied to show that the formation of MCCPs, as the major byproduct of this method, would increase not only at longer heating durations, but also as a result of higher heating temperatures. The MCCPs were shown to have a negative influence on PL of the solution, whose effect can be minimized by diluting the sample.

Finally, 160–50 sample was employed for detection of Hg^2+^, showing high selectivity and sensitivity, with detection limit of 50 nM. A facile and cheap smartphone sensing platform was designed and employed, which is capable of quantifying and comparing PL intensities of several samples simultaneously.
